# Fine mapping of a Fusarium crown rot resistant locus on chromosome arm 6HL in barley by exploiting near isogenic lines, transcriptome profiling, and a large near isogenic line-derived population

**DOI:** 10.1007/s00122-023-04387-x

**Published:** 2023-05-26

**Authors:** Shang Gao, Yunfeng Jiang, Hong Zhou, Yaxi Liu, Huihui Li, Chunji Liu, Zhi Zheng

**Affiliations:** 1 CSIRO Agriculture and Food, 306 Carmody Road, St Lucia, QLD 4067 Australia; 2 Institute of Crop Sciences, Chinese Academy of Agricultural Sciences, CIMMYT-China Office, 12 Zhongguancun South Street, Beijing, 100081 China; 3 Nanfan Research Institute, Chinese Academy of Agricultural Sciences, Sanya, 572024 Hainan China; 4 Triticeae Research Institute, Sichuan Agricultural University, Wenjiang, Chengdu, 611130 China

## Abstract

**Key message:**

This study reported validation and fine mapping of a Fusarium crown rot resistant locus on chromosome arm 6HL in barley using near isogenic lines, transcriptome sequences, and a large near isogenic line-derived population.

**Abstract:**

Fusarium crown rot (FCR), caused by *Fusarium pseudograminearum*, is a chronic and serious disease affecting cereal production in semi-arid regions globally. The increasing prevalence of this disease in recent years is attributed to the widespread adoption of minimum tillage and stubble retention practices. In the study reported here, we generated eight pairs of near isogenic lines (NILs) targeting a putative QTL (*Qcrs.caf-6H*) conferring FCR resistance in barley. Assessing the NILs confirmed the large effect of this locus. Aimed to develop markers that can be reliably used in incorporating this resistant allele into breeding programs and identify candidate genes, transcriptomic analyses were conducted against three of the NIL pairs and a large NIL-derived population consisting of 1085 F7 recombinant inbred lines generated. By analyzing the transcriptomic data and the fine mapping population, *Qcrs.caf-6H* was delineated into an interval of 0.9 cM covering a physical distance of ~ 547 kb. Six markers co-segregating with this locus were developed. Based on differential gene expression and SNP variations between the two isolines among the three NIL pairs, candidate genes underlying the resistance at this locus were detected. These results would improve the efficiency of incorporating the targeted locus into barley breeding programs and facilitate the cloning of causal gene(s) responsible for the resistance.

**Supplementary Information:**

The online version contains supplementary material available at 10.1007/s00122-023-04387-x.

## Introduction

Fusarium crown rot (FCR) is a widespread and damaging disease affecting the production of many crop species including wheat, barley, durum, oat, and triticale in semi-arid regions worldwide. The prevalence of FCR has increased in conservation cropping systems in recent years (Paulitz et al. 2002; Smiley et al. 2005; Saremi et al. 2007; Tunali et al. 2008; Chakraborty et al. 2010; Hameed et al. 2012; Li et al. 2016; Xu et al. 2017) likely due to the tight rotations of cereal crops and the retention of stubble (Simpfendorfer et al. 2019). FCR damage in barley has been overlooked due to its less yield loss compared to wheat (Smiley et al. 2005; Daniel and Simpfendorfer 2012). This is likely due to the fact that whiteheads, a commonly used symptom to evaluate FCR severity in wheat, are rarely present in infected barley plants. Despite this, more severe disease symptoms and higher accumulation of *Fusarium* mycelia were observed in barley when compared to wheat at similar stages of FCR infection (Liu et al. 2012a). *Fusarium* mycelia can colonize stubble residue and persist for up to three years or more (Burgess 2014), which increases the risk of FCR infection in the following crop seasons. Therefore, developing barley varieties with enhanced FCR resistance would not only reduce grain yield loss in barley itself but might also benefit subsequent crops by reducing the inoculum load in stubbles.

Understanding the genetics of resistant sources is crucial for breeding programs against FCR (Liu and Ogbonnaya 2015). To achieve this, quantitative trait locus (QTL) mapping has been routinely adopted to detect resistance loci. To date, four putative QTL have been detected in barley and their effects were consistently expressed in different genetic backgrounds. They were located on chromosome arms 1HL (Chen et al. 2013b), 3HL (Li et al. 2009), 4HL (Chen et al. 2013a), and 6HL (Gao et al. 2019a; Gao 2020), respectively. However, morphological traits such as plant height and heading date have been found to have a passive contribution to FCR resistance (Liu et al. 2012a, 2012b; Chen et al. 2013b; Bai and Liu 2015). When the effects of these morphological traits were removed by covariance analysis, both LOD values and magnitudes of the QTL were reduced (Chen et al. 2013a; Zheng et al. 2014). As a result, any loci identified from QTL studies must be treated as putative. To accurately assess the effects of a locus and to develop reliable markers targeting an FCR locus, segregation of those undesired characteristics needs to be fixed.

Development of near isogenic lines (NILs) has been considered one of the most effective approaches to achieve fixed genetic backgrounds and has been routinely used to validate FCR resistant loci in wheat and barley by our team (Ma et al. 2012; Habib et al. 2016; Gao et al. 2020; Gao 2020). The difference between the two isolines of a NIL pair is primarily limited in the targeted region, facilitating further investigation on elucidating potential mechanisms via transcriptomic analyses. RNA sequencing (RNA-seq) has become an indispensable tool for transcriptomic profiling (Wang et al. 2009; Stark et al. 2019) and has been employed to understand the mechanisms of various diseases. Compared to traditional studies on genetically distinct genotypes which differ significantly in their genetic backgrounds, analyzing NILs offer huge advantages including minimization of genetic background interference and increased sensitivity and accuracy of transcriptional analyses (Keurentjes et al. 2007). Moreover, populations derived from the NILs primarily segregating at a targeted locus can also convert a quantitative trait into a Mendelian factor facilitating the accurate localization of a locus (Zheng et al. 2015; Jiang et al. 2019; Gao et al. 2020).

In the study reported here, we aimed to validate the effect of the putative QTL (*Qcrs.caf-6H*) conferring FCR resistance on chromosome arm 6HL in barley (Gao et al. 2019a, b) and explore its underlying mechanism. A few pairs of NILs were developed to minimize genetic background interference and improve the accuracy of the transcriptional analysis. Transcriptomic sequences were obtained from three of these NIL pairs and used to identify single nucleotide polymorphism (SNP) variations and candidate genes. To develop markers tightly linked with this locus, a large NIL-derived population consisting of 1085 F7 lines was then generated and assessed. Taken together, markers co-segregating with this locus were generated and a small number of candidate genes underlying *Qcrs.caf-6H* were identified in the present study.

## Materials and methods

### Plant materials

Two segregating populations generated by crossing a resistant donor AWCS799 and two Australian varieties Fleet and Franklin were used in this study (Gao et al. 2019a). AWCS799 was identified from a screening of 1,047 genotypes representing different geographical origins and plant types (Liu et al. 2012b). Combined with a fast generation procedure (Zheng et al. 2013), the method of heterogeneous inbred family (HIF) (Tuinstra et al. 1997) was used to develop NILs in glasshouses at Queensland Bioscience Precinct (QBP), Brisbane. An SSR marker, *6H_497772849* (forward primer GCATTAGTTGTCATAGTAGGTAGCA and reverse primer TTCAAGACCACGACCTTGGG) which was closely linked with *Qcrs.caf-6H* (Gao et al. 2019a), was used to identify heterozygous plants from each of the two populations. Heterozygous plants identified were self-pollinated, and ten plants derived from each of the heterozygous plants were used for the next round of selection. This process of selecting heterozygous individuals and self-pollination was repeated until F8 generation. Two isolines, one with the resistant allele and the other with the susceptible allele, were then isolated from each of the F8 heterozygous plants and were treated as a pair of putative NIL. Seeds from these putative NIL pairs were then increased at Gatton Research Station for further assessments.

A NIL-derived population consisting of 1085 lines was generated as described above from four different heterozygous F6 plants obtained in generating the 6HL_NIL1 which showed the biggest difference on disease index between R and S isolines. A single-seed-descent approach was used to process this population to F7 generation. Seeds from each of the lines were then increased in glasshouses at QBP.

### Assessment of FCR resistance

Previous studies indicated that FCR resistance is unlikely to be species-specific (Van Eeuwijk et al. 1995; Chakraborty et al. 2010; Li et al. 2010b; Ma et al. 2010). Therefore, one highly aggressive isolate of *F. pseudograminearum (Fp)* CS3096 (Gao 2020) was used to assess FCR resistance in this study. Protocols for inoculum preparation, FCR inoculation, and disease severity measurement were conducted following the methods described by Li et al. (2008). In brief, a piece of infected plant fragment was placed on plates containing 1/2 strength potato dextrose agar (PDA). Inoculated plates were incubated at room temperature for 14 days, and the mycelium was then removed. The plates were further incubated for another 14 days under a combination of cool white and black fluorescent lights with a 12-h photoperiod. Spores were harvested, and the concentration of spore suspension was adjusted to 1 × 10^6^ spores/ml. The spore suspension was stored in a – 20 °C freezer, and Tween 20 (0.1%v/v) was added before use.

Seeds were germinated on two layers of filter paper saturated with water in Petri dishes. The Petri dishes were chilled in a cold room at 4 °C for two days for uniform germination and then, were left at room temperature overnight before transferring into potting mix. The seedlings with similar lengths (~ 0.5 cm) were immersed in the spore suspension for 2 min. Two seedlings were planted into each square punnet (80 cm^3^) of a 56-well tray (Rite Grow Kwik Pots, Garden City Plastics, Australia) containing sterilized University of California mix C (50% sand and 50% peat v/v). The trays were arranged in a randomized block design and placed in a controlled environment facility (CEF) at QBP. The CEF was set at 25/18 (± 1) °C Day/night temperature, 65/80% (± 5) % day/night relative humidity, and a 14 h photoperiod with 500 µmol m^−2^ S^−1^ photon flux density at the level of the plant canopy (Gao 2020). As drought stress would promote FCR development (Liu and Liu 2016), the inoculated seedlings were watered during the assessment only when wilt symptoms were detected.

Four independent trials were conducted to assess FCR resistance against the putative NILs and a subpopulation of 96 NIL-derived lines, while six trials were performed against recombinants identified from the whole NIL-derived population of 1,085 lines. The resistant and susceptible isolines of the NIL_CR6HL_1 were used as positive and negative controls in each of the trials. Each trial contained three replicates and each replicate consisted of 14 seedlings. The flanking markers of the *Qcrs.caf-6H* locus were determined using the subpopulation and used to identify recombinant lines in the whole NIL-derived population. FCR severity was evaluated four weeks after inoculation using a 0–5 scale, where ‘0’ representing no symptom and ‘5’ standing for whole plant necrotic (Li et al. 2008). Disease indices (DI) were calculated for each line following the formula of DI = (Σ_*nX*_ /5 *N*) × 100, where *X* is the scale value of each plant, *n* is the number of plants in the category, and *N* is the total number of plants assessed for each line.

### Statistical analysis

Statistical analyses were performed using the SPSS statistics 19.0 for Windows statistical software package (SPSS Inc., Chicago, IL). For each trial, the following mixed-effect model was used: *Yij* = *μ* + *ri* + *gj* + *wij*, where: *Yij* = trait value on the *j*th genotype in the *i*th replication; *μ* = general mean; *ri* = effect due to *i*th replication; *wij* is the error or genotype by replication interaction, where genotype was treated as a fixed effect and that of replicate as random. The Tukey’s HSD test of one-way ANOVA was employed to detect possible differences among the means. The disease ratings from all seedlings for each line of the fine mapping population in each trial were averaged and used to determine whether the line in concern was resistant (< 2.5) or susceptible (> 2.5) to FCR infection (Gao 2020).

### RNA extraction and sequencing

Samples for RNA sequencing were collected from three pairs of the NILs (namely 6HL_NIL1, 6HL_NIL2, and 6HL_NIL3). Inoculation was conducted with either the *F. pseudograminearum* isolate (*Fp*-inoculation) or distill water (mock) following the protocol described by Gao et al. (2020). Three biological replications were conducted for each of the isolines and each replication contained 14 seedlings. Tissues for RNA extraction were obtained by cutting the shoot bases (2 cm) of 14 seedlings at 4 days post-inoculation (dpi) and snap-frozen in liquid nitrogen and kept at − 80 °C until processed. The time point for sampling was based on previous studies (Habib et al. 2018; Gao et al. 2019b; Gao 2020).

A total of 36 samples were obtained from the six isolines. Samples were crushed into fine powder in a 1.5 μL micro-centrifuge tube and total RNA was extracted using a RNeasy plant mini kit (Qiagen, Hilden, Germany) according to manufacturer’s instructions (including DNase-I digestion). The yield and purity of RNA samples were measured using a Nanodrop-1000 Spectrophotometer. The integrity of all RNA samples was assessed by running the total RNA on 1% agarose gels. RNA sequencing was carried out by the Australian Genome Research Facility Ltd. (Parkville, Victoria, Australia), and 100-bp paired-end reads were produced using the Illumina Hiseq-2000. All 36 RNA-seq libraries were run across four lanes of a HiSeq2000. The RNA sequencing files have been made available at the National Center for Biotechnology Information (NCBI) with the accession number PRJNA922199.

### Transcriptomic analysis

Commands used for trimming raw data and analyzing trimmed reads were described by Habib et al. (2018). FastQC (version 0.11.2) was used as a preliminary check for PHRED scores. Raw reads were trimmed using the SolexaQA package (version 2.0.13) with a minimum PHRED quality value of 30 and a minimum final read length of 70 bp. The filtered reads were then mapped to the reference genome of Morex (https://webblast.ipk-gatersleben.de/barley_ibsc/downloads/:150831_barley_pseudomolecules) using BWA (version 0.7.12) (Li and Durbin 2009).

### Analysis of differential gene expression

Cufflinks v2.0.2 (Roberts et al. 2011) was used to assemble the mapped reads. Differentially expressed genes (DEGs) were identified with Cuffdiff from the Cufflinks tool package with high-confidence genes annotated from the ‘Morex’ reference. Fragments per kilobase of exon per million mapped reads (FPKM) were applied for each transcript to represent the normalized expression value. The fold change was calculated according to the equation: Fold Change = log_2_ (FPKM_*A*_/FPKM_*B*_).

Pairwise comparisons between different treatments for the same genotype and between genotypes under *Fp-*inoculation and mock-inoculation were conducted (Fig. S1). These are summarized throughout the paper in the following way S^M^_v_S^I^, R^M^_v_R^I^, S^I^_v_R^I^ and S^M^_v_R^M^, where ‘M’ for ‘Mock’; ‘I’ for *Fp*-inoculation; ‘S’ for the susceptible isoline; and ‘R’ for the resistant isoline. DEGs were determined with the adjusted *p*-value threshold of ≤ 0.05 and log_2_ expression fold change of ≥ 1 or ≤ -1 or ‘inf’ (where the FPKM value in one condition is zero, and the other is not). Venny 2.0 was used for Venn diagram analysis (Oliveros 2007).

### SNP calling and marker development

A graphical overview of the experimental design for SNP calling has been provided (Fig. S1). All six sequence files (three biological replicates by two treatments) for each genotype were concatenated after removing low-quality sequences. The files were then aligned to the Morex genome using BWA align. Duplicates were removed by SAMtools rmdup and SNPs were identified using SAMtools mpileup by skipping alignments with mapQ smaller than 20, then SNPs with DP < 4 and SNPs within 3 base pairs of an indel were filtered by SAMtools/BCFtools (version 1.5) (Li 2011). The variant effect of SNPs in candidate genes was evaluated with snpEff (Version 5.1) (Cingolani et al. 2012). Kompetitive allele specific PCR (KASP) markers were then developed based on the SNPs detected. All the primers were designed using the Primer-BLAST (Ye et al. 2012). Linkage analysis was carried out using the computer package JoinMap 4.0 (Van Ooijen 2006).

### DNA extraction and genotyping

Leaf tissue from each line of the NIL-derived population was collected and vacuum dried for DNA extraction using the CTAB protocol (Porebski et al. 1997). KASP assay was conducted using 384-well set on the Vii 7 Real-Time PCR system (Applied Biosystems, Foster City, California, USA). One KASP reaction volume consisted of a total volume of 5 μl including 2.0 μl DNA (20 ng/μl), 0.06 μl primer mixture, and 3 μl of KASP Master Mix. On each SNP reaction plate, at least one water sample was included as a no-template control. The PCR profile started with an initial denaturation step at 94 °C for 15 min, continued with 10 touch-down PCR cycles at 94 °C for 20 s, and 60 °C for 1 min with − 0.5 °C/cycle, and then 30 cycles at 94 °C for 20 s, and 55 °C for 1 min. Three replicates for each genotype were performed.

### Validation of differentially expressed genes

Expression assessments of five candidate genes in the targeted interval (primer sequences were listed in Table S1) selected from transcriptomic analyses were conducted using quantitative real-time PCR (qRT-PCR) with the actin protein gene as the internal housekeeping reference (forward primer: 5′-GCCGTGCTTTCCCTCTATG-3′; reverse primer 5′-GCTTCTCCTTGATGTCCCTTA-3′). Inoculation, tissue sampling, and RNA extraction were carried out using the aforementioned methods. Three biological replicates, each with two technical replications, were used for each genotype-treatment sample per isoline.

The procedures for synthesizing cDNA and qRT-PCR were described by Gao et al. (2019a, b). The relative fold changes were calculated using the comparative CT method (2^−ΔΔCT^). Average values of the two technical replications were used to represent the biological replicate for each of the samples.

## Results

### Near isogenic lines targeting the Qcrs.caf-6H locus

**Fig. 1:**
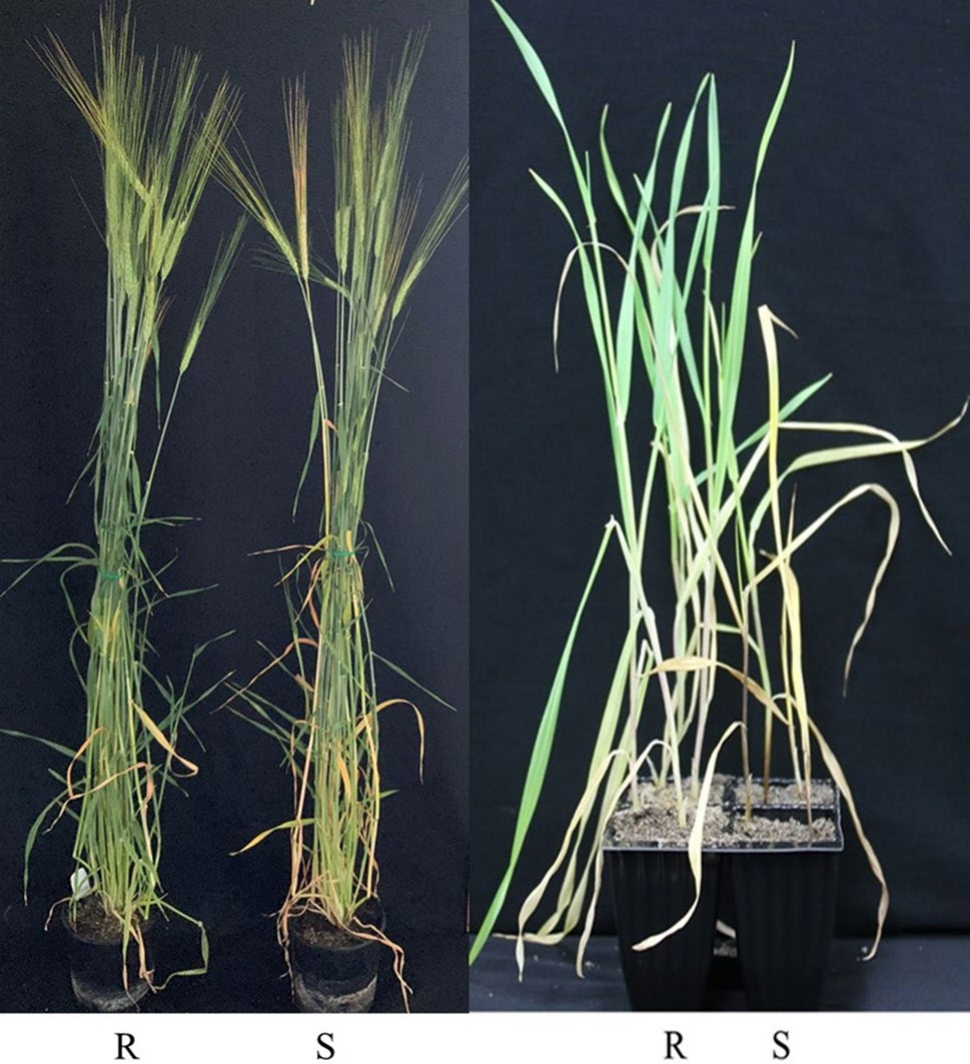
Plants of a pair of the near isogenic lines (NIL_CR6HL_1R and NIL_CR6HL_1S) showing similar morphology of the non-inoculated plants (left) and the difference in resistant to *Fusarium pseudograminearum* at 28 days post-inoculation (right). *R* represents lines with the resistant allele, and *S* represents lines without the resistant allele

Eleven pairs of putative NILs were initially developed to validate the effect of the 6HL resistant locus based on the marker profiles of *6H_497772849*. Among them, eight pairs showed significant differences in disease severity between the two isolines (Fig. 1) with no clear morphologic differences. The resistant isolines performed better on FCR resistance with an average DI value of 34.2 compared with the susceptible isolines with an average DI value of 50.2 (Table 1).

**Table 1:** Difference in disease index between the resistant and susceptible isolines for eight NIL pairs targeting the *Qcrs.caf-6H* conferring FCR resistance

NILs	Genetic backgrounds	DI Mean	Difference (%)	*P* value
6HL_NIL1R	Fleet/AWCS799	32.8	41.8	< 0.01
6HL_NIL1S		56.4		
6HL_NIL2R	Fleet/AWCS799	32.4	39.1	< 0.01
6HL_NIL2S		53.1		
6HL_NIL3R	Fleet/AWCS799	31.9	31.3	< 0.01
6HL_NIL3S		46.5		
6HL_NIL4R	Fleet/AWCS799	33.6	34.8	< 0.01
6HL_NIL4S		51.6		
6HL_NIL5R	Franklin/AWCS799	35.7	28.8	< 0.01
6HL_NIL5S		52.3		
6HL_NIL6R	Franklin/AWCS799	37.3	27.2	< 0.01
6HL_NIL6S		48.4		
6HL_NIL7R	Franklin/AWCS799	34.4	27.7	< 0.01
6HL_NIL7S		47.7		
6HL_NIL8R	Franklin/AWCS799	35.3	21.4	< 0.01
6HL_NIL8S		45.4		

‘R’ lines represent those with allele from the resistant parent AWCS799, and ‘S’ lines are those with an alternative allele from the susceptible parents; DI mean represents the mean of disease index from three trials for each isoline

### DEGs in the Qcrs.caf-6H region

**Fig. 2:**
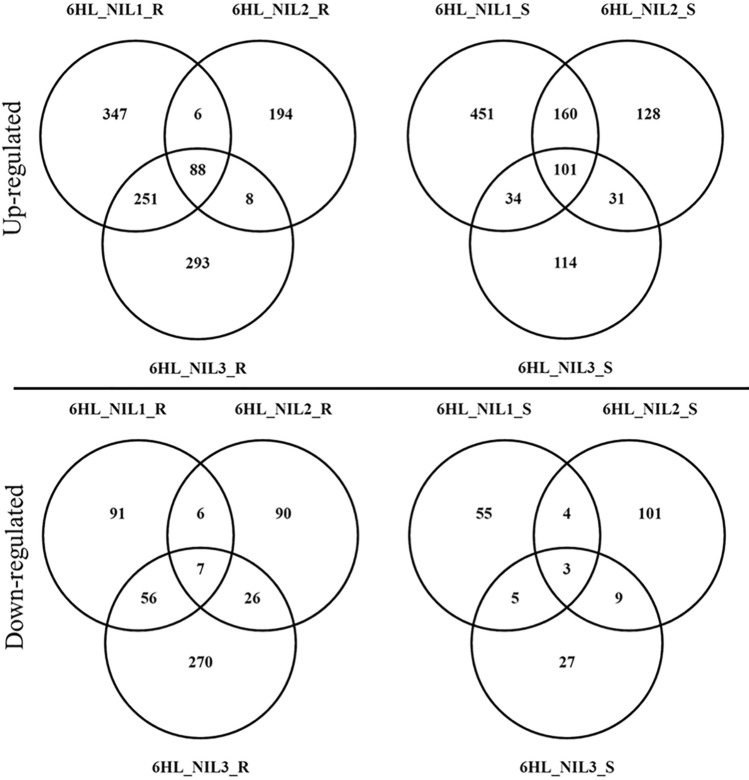
DEGs for each of the 6HL_NIL pairs following *Fp*- and mock-inoculation (R^M^_vs_R^I^) and S^M^_vs_S^I^). Venn diagrams in upper panel show the numbers of up-regulated DEGs in each ‘R’ (left) and ‘S’ (right) isolines. Venn diagrams in lower panel show the numbers of down-regulated DEGs in each ‘R’ (left) and ‘S’ (right) isolines. DEGs were determined with the threshold of FDR ≤ 0.05 and |log2 fold change|≥ 1 or ‘inf’ (one of the comparative objects did not express and the other did)

Three of the eight NIL pairs with the largest differences in FCR severity were selected and used to generate RNA-seq. To analyze host response to FCR infection, DEGs were detected between *Fp*- and mock-inoculated samples of the same isolines. The numbers of up-regulated genes were significantly higher than those of down-regulated ones following *Fp*-inoculation (Table S2). Of the up-regulated genes, 88 were shared by all the three ‘R’ isolines while 101 were shared by the three ‘S’ isolines (Fig. 2). Of the down-regulated genes, only seven were shared among the three ‘R’ isolines and three among the ‘S’ isolines (Fig. 2).

**Fig. 3:**
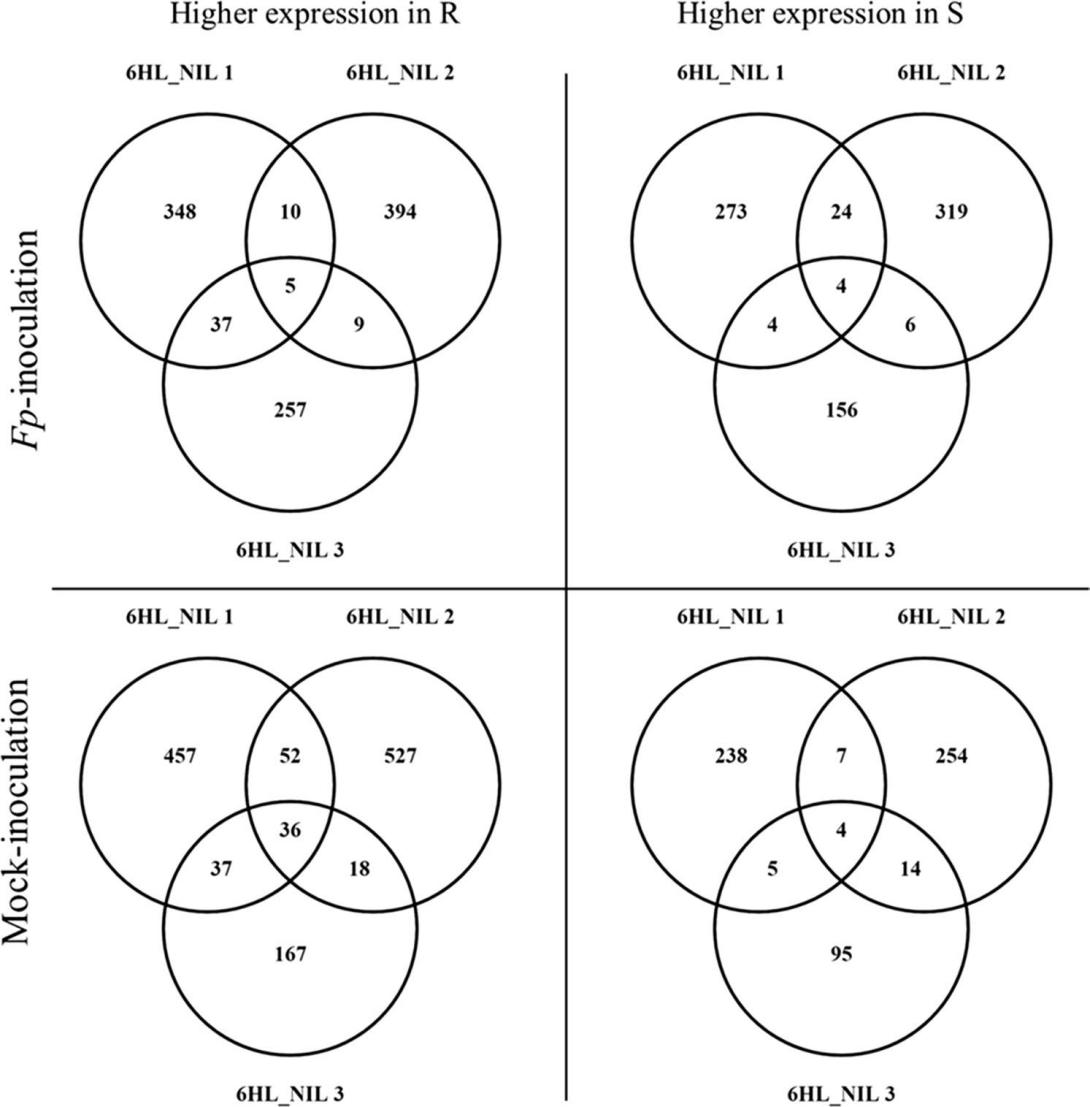
DEGs between ‘R’ and ‘S’ isolines under *Fp*- (R^I^_vs_S^I^) or mock-inoculation (R^M^_vs_S^M^). Venn diagrams show the numbers of DEGs up-regulated in ‘R’ (left) or ‘S’ (right) isolines under *Fp*- (up) or mock-inoculation (down). DEGs were determined with the threshold of FDR ≤ 0.05 and |log2 fold change|≥ 1 or ‘inf’ (one of the comparative objects did not express and the other did)

To assess transcriptomic responses to FCR infection mediated by *Qcrs.caf-6H*, DEGs between the ‘R’ and ‘S’ isolines were also compared. These comparisons detected a total of 1060 up-regulated genes and 786 down-regulated ones from the *Fp*-treatment (Table S2). Only five of the up-regulated genes and four of the down-regulated ones were shared by all three NIL pairs, respectively (Fig. 3). Of the DEGs identified from the mock-inoculated samples, there were 1294 up-regulated genes which were more than twice of the down-regulated ones (Table S2). Among them, 36 up- and four down-regulated genes overlapped across all three comparisons (Fig. 3).

### SNPs between the ‘R’ and ‘S’ isolines shared with three 6H_NIL pairs

A total of 37,972 homozygous SNPs were detected between isolines for the three NIL pairs. The number of SNPs detected in the NIL_CR6HL_3 was more than twice compared to those detected from either of the other two NIL pairs (Fig. S2). This number was dramatically reduced to 278 when comparing the R and S isolines among three NIL pairs (Fig. S2) and all of them fell into the distal end of the chromosome arm 6HL obtaining the *Qcrs.caf-6H* locus spanning a physical interval of ~ 22 Mbp (Fig. S3a). Based on the reference genome of barley cv. Morex, 142 high-confident (HC) genes and 150 low-confident (LC) genes were identified in the common interval across three 6HL NIL pairs. Among these HC genes, 30 contained SNP variants shared by all three NIL pairs and eight were differentially expressed between the isolines for at least one of the NIL pairs (Fig. S3b).

### Chromosomal interval containing Qcrs.caf-6H based on the subpopulation

**Fig. 4:**
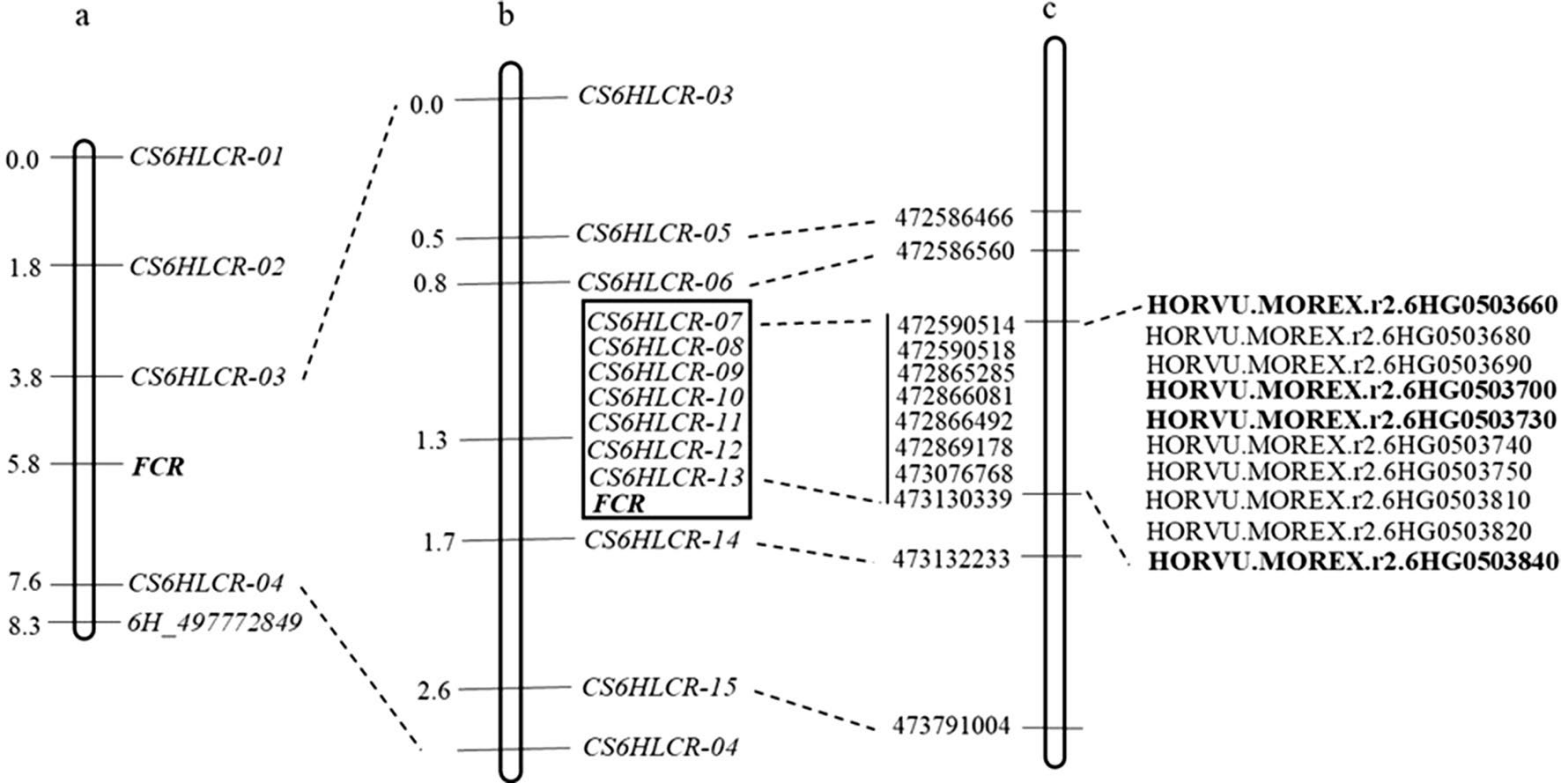
Genetic and physical maps surrounding the Fusarium crown rot resistant locus *Qcrs.caf-6H* in barley. **a** The targeted interval based on the assessment of a subpopulation consisting of 96 NIL-derived lines; **b** The high-density linkage map around the targeted locus based on the analysis of 1,085 NIL-derived lines. Markers co-segregating with the FCR locus were placed in the box; **c** physical locations of marker on the 6H pseudomolecule and high-confident genes detected in the targeted interval. DEGs detected between ‘R’ and ‘S’ isolines for at least one of the NIL pairs in the region were highlighted in bold

Four polymorphic markers in the targeted interval were developed based on sequence differences between the resistant and susceptible isolines. Together with *6H_497772849*, the five markers were used to screen the sub-populations consisting of 96 lines (Fig. 4a). Linkage analysis showed that they spanned a genetic distance of 8.3 cM. Disease ratings of this subpopulation fell into two classes: one rating from 18.4 to 37.2 (resistant) and the other from 54.9 to 78.6 (susceptible). There were no intermediate ratings for any of the lines assessed (Fig. S4). After analyzing the marker profiles and the phenotypic data, *Qcrs.caf-6H* was mapped into a 3.8 cM interval flanked by *CS6HLCR-03* and *CS6HLCR-04* (Fig. 4a).

### Fine mapping of Qcrs.caf-6H locus using the whole NIL-derived population

**Fig. 5:**
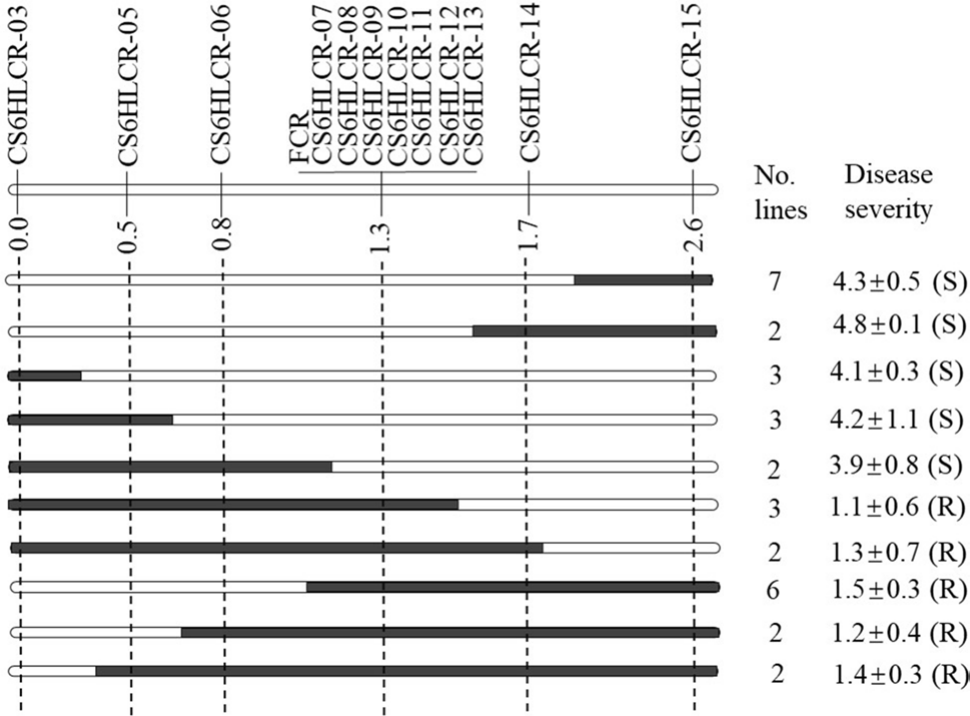
Genotypes and phenotypes of the haplotypes identified from the recombinant lines based on markers surrounding the *Qcrs.caf-6H* locus. The solid regions represent alleles from the resistant parent and the empty ones for the susceptible parent. Phenotypic values (mean ± SE) were calculated based on the disease severity from all trials. The numbers on the chromosome stand for genetic distance (cM)

A total of 32 recombinants were identified among the whole fine mapping population of 1,085 lines using the two flanking markers. These recombinants were categorized into either resistant or susceptible groups, with a significant difference between the two groups (*P* < 0.01). Of the 32 recombinants, 15 lines were classified as resistant, while the remaining 17 lines were susceptible (Fig. 5).

To further narrow down the candidate region, eleven KASP markers were newly generated based on the SNP calling results and were found to be polymorphic between the two isolines. Genotyping the recombinants revealed that six of the eleven KASP markers co-segregated with the *Qcrs.caf-6H* locus (Table 2), mapping the locus into a 0.9 cM interval between *CS6HLCR-07* and *CS6HLCR-13* (Fig. 4b). These markers were found to be highly consistent with their relative physical positions around the targeted region (Fig. 4b, c), with the *Qcrs.caf-6H* locus falling into a ~ 547 kb interval between 472.58 and 473.13 Mbp on the reference genome of barley cv. Morex (Fig. 4c).

**Table 2:** Primer sequences for KASP markers co-segregating with the *Qcrs.caf-6H*

Marker	Physical position	Allele	Forward primer (5′–3′)	Common reverse primer (5′–3′)	*T*_m_ (°C)
*CS6HLCR-07*	472,590,514	R	GAAGGTGACCAAGTTCATGCTGAATCAGGCGGGTGGTTGA**T**	GCAGAAGCAGGTCGAGGTAC	60
		S	GAAGGTCGGAGTCAACGGATTGAATCAGGCGGGTGGTTGA**C**		
*CS6HLCR-08*	472,590,518	R	GAAGGTGACCAAGTTCATGCTCAGGTCGAGGTACGCATCA**C**	ACGAGAGAAGCGGAATCAGG	60
		S	GAAGGTCGGAGTCAACGGATTCAGGTCGAGGTACGCATCA**T**		
*CS6HLCR-09*	472,865,285	R	GAAGGTGACCAAGTTCATGCTCCTTGAAGATTATCTGTGACCTTT**C**	ACGAAGCTTCCTTCTGCCAA	60
		S	GAAGGTCGGAGTCAACGGATTCCTTGAAGATTATCTGTGACCTTT**T**		
*CS6HLCR-10*	472,866,081	R	GAAGGTGACCAAGTTCATGCTCAGTACCTCTAATTGCTGGGAAT**G**	GCATCAGAGAACGCGACAGA	60
		S	GAAGGTCGGAGTCAACGGATTCAGTACCTCTAATTGCTGGGAAT**T**		
*CS6HLCR-11*	472,866,492	R	GAAGGTGACCAAGTTCATGCTTGTGGCACTTTTCAGGAGTAC**T**	ACACTGGTGAGCGGAATGAA	60
		S	GAAGGTCGGAGTCAACGGATTTGTGGCACTTTTCAGGAGTAC**A**		
*CS6HLCR-12*	472,869,178	R	GAAGGTGACCAAGTTCATGCTCTTGGTTTTGGGTCGTTCCT**A**	GACGAGACAACCAACCTGCT	60
		S	GAAGGTCGGAGTCAACGGATTCTTGGTTTTGGGTCGTTCCT**C**		
*CS6HLCR-13*	473,130,339	R	GAAGGTGACCAAGTTCATGCTCGAAAGTTTCCATTGCCAGG**T**	CGACAGAGATGCAGAGCTAGG	55
		S	GAAGGTCGGAGTCAACGGATTCGAAAGTTTCCATTGCCAGG**A**		

Allele-specific nucleotides in the forward primers are in bold

### Identification of candidate genes in the targeted region

Ten HC and eight LC genes were identified in the refined interval (Fig. 4c, Table S3). Because the LC genes lacked clear functional annotations, they were not considered in further analysis. Among the HC genes, five of them contain SNP variants between resistant and susceptible isolines shared by all the three NIL pairs (Fig. 4c, Table S4). Expression patterns consistent with the RNA-seq analysis were obtained in the qRT-PCR analysis for each of the five genes assessed (Table S1). Of the five genes, it is of interest to note that only *HORVU.MOREX. r2.6HG0503730,* coding a U-box domain-containing family protein, harbors three missense variants leading to amino acid changes (Table S4). Moreover, this gene was *Fp*-induced down-regulated in two of the three analyzed susceptible isolines. These results indicate that the U-box protein coding gene is likely to be a strong candidate underlying *Qcrs.caf-6H* locus.

## Discussion

FCR is a devastating threat to cereal production worldwide, particularly in semi-arid regions. In efforts to address this challenge, breeding varieties with boosted resistance are critical. Working toward this, sources of FCR resistance have been identified and putative QTL conferring resistance was detected. In this study, we aimed to develop markers that can be reliably used to tag the 6HL resistant locus for further incorporating this locus into breeding programs. Resources including eight pairs of NILs, RNA sequences targeting three of the NIL pairs, and a large NIL-derived population were generated and assessed. Using markers developed from the RNA-seq analysis, *Qcrs.caf-6H* was successfully placed into a 0.9 cM interval covering a physical distance of approximately 547 kb. A candidate gene coding the U-box domain-containing family protein was identified harboring three missense variants between the two isolines, which would be the primary target in further investigations on gene cloning.

In this study, eleven pairs of NILs were initially generated. However, no significant difference in FCR resistance was detected between the two isolines for three of the eleven pairs of putative NILs. Different from the method of using flanking markers for the targeted locus (Pumphrey et al. 2007), we generated NILs using only one linked marker which should reduce the sizes of chromosomal segments differentiating the isolines (Ma et al. 2012; Habib et al. 2016; Gao et al. 2020). However, markers obtained from QTL mapping studies may not be tightly linked with a targeted locus due to the limited resolution (Paterson et al. 1988). It is clear recombination between the linked and its target may occur resulting in false NILs. In the present study, significant variation was also found in the numbers of SNPs and DEGs detected among the three pairs of NILs assessed as different NIL pairs were expected to have different genetic backgrounds which potentially caused such variation and would lead to the difference in FCR development at any given time point. While individual NIL pair contribute large sets of DEGs and SNPs, analysis of multiple NIL pairs could detect conserved SNP differences. This approach, which would not only be effective in narrowing the candidate regions and reducing the number of candidate genes but also uncover genetic markers for fine mapping the targeted loci, has been routinely used for FCR studies in our team (Ma et al. 2014; Habib et al. 2018; Jiang et al. 2019; Gao et al. 2019b, 2020).

Previous studies reported the accuracy of FCR assessment can be affected by several morphologic characteristics including plant height and heading date (Liu et al. 2012a, 2012b; Chen et al. 2013b). Thus, a large NIL-derived population was subsequently developed to minimize the interference from the segregations of these non-targeted characteristics. The individuals in this population mainly differed in the targeted region, allowing precise categorization of FCR severity into either resistant or susceptible. As a result, *Qcrs.caf-6H* was placed into a refined region containing ten HC genes (Fig. 4). However, none of these genes is a classic NBS-LRR (nucleotide-binding sites and leucine-rich-repeat) resistant gene. Previous studies showed that non-classical NBS-LRR genes confer resistance to *Fusarium* head blight (FHB), another disease caused by *Fusarium* pathogens (Moore et al. 2015; Ke et al. 2017; Milne et al. 2019). Similarly, host resistance to FCR and FHB is not pathogen species-specific (Fv et al. 1995; Chakraborty et al. 2010; Li et al. 2010b; Ma et al. 2010). Thus, it is likely that non-classical NBS-LRR genes may also be responsible for FCR resistance.

Of the ten HC genes located within the targeted interval, four were differentially expressed bearing SNP variations between the two isolines among all the three NIL pairs. They were related to either or both biotic and abiotic stresses. Among them, *HORVU.MOREX. r2.6HG0503730* coding a U-box domain-containing family protein is the only one containing missense variants, which would form our primary target. The functions of this gene in biotic and abiotic stress are well known (Yan et al. 2003; Zeng et al. 2004) including its role in pathogen recognition and plant immune response to broad-spectrum disease resistance to various pathogens (González-Lamothe et al. 2006; Cheng and Li 2012; Li et al. 2012). Although synonymous variants do not alter the encoded amino acids, they can still alter mRNA structure leading to changes in the rate or efficiency of translation (Hunt et al. 2014) and/or protein production (Komar 2016) and abundance (Maier et al. 2009), which in turn impact fitness (Lebeuf-Taylor et al. 2019; Bailey et al. 2021). Thus, the other three DEGs in the candidate region cannot be ruled out. The second gene *HORVU.MOREX. r2.6HG0503700* encodes a basic helix-loop-helix (bHLH) transcription factor which has been functionally characterized in many plant species to contribute to immunity against various biotic and abiotic stresses (Heim et al. 2003; Li et al. 2010a). Previous studies showed that bHLH functions as a transcriptional activator associated with NBS-LRR genes to activate defense responses (Xu et al. 2014). The third gene *HORVU.MOREX. r2.6HG0503660* encodes an Alpha/Beta hydrolase fold protein, which has been reported associated with defense against pathogens (Wäspi et al. 1998) and abiotic stress such as salinity and drought (Liu et al. 2014; Akbudak et al. 2019). In addition, it has been reported that the severity of FCR can be strongly affected by drought stress (Liu and Liu 2016). Thus, the fourth gene *HORVU.MOREX. r2.6HG0503840* encoding ATPase family AAA domain-containing protein 3 relevant to abiotic stresses (Kim et al. 2021) should be considered as another candidate for FCR resistance at this locus.

## Supplementary Information

Below is the link to the electronic supplementary material.

Supplementary file 1.

## Data Availability

The datasets generated during and/or analyzed during the current study are available from the corresponding author upon reasonable request.
