# Supplementary material for: Fine mapping of a Fusarium crown rot resistant locus on chromosome arm 6HL in barley by exploiting near isogenic lines, transcriptome profiling, and a large near isogenic line-derived population
Source: Theor Appl Genet. 2023 May 26;136(6):137. doi: 10.1007/s00122-023-04387-x (PMC10220118; doi:10.1007/s00122-023-04387-x)
Supplement: Supplementary file 1 [file 122_2023_4387_MOESM1_ESM.docx]

Table S1 Primer sequences and qRT-PCR relative expression patterns of five genes selected for validation of RNA-seq results.

| Gene ID | Primer 5’ -> 3’ | Comparison | log₂FC Cuffdiff | log₂FC qPCR |
| --- | --- | --- | --- | --- |
| HORVU.MOREX.r2.6HG0503660 | Forward:ATACGCACGGCAACAAGTTTC | 2R_vs_2S_Fp | -1.97 | -2.30 |
|  | Reverse:GGATGTTGTACCTCCAATGG |  |  |  |
| HORVU.MOREX.r2.6HG0503700 | Forward:GACGACAAGTACCGCGCAT | 1R_vs_1S_mock | -2.51 | -1.96 |
|  | Reverse:TGGATGTAGTCGGTCTTCTG | 3R_vs_3S_Fp | -2.17 | -1.98 |
| HORVU.MOREX.r2.6HG0503730 | Forward:CTGGGAATAATCTCGAAGCTG | Fp_vs_mock_1S | -2.10 | -2.53 |
|  | Reverse:AGCTTGAACGAGCAACTCTTC | Fp_vs_mock_2R | -1.90 | -2.82 |
| HORVU.MOREX.r2.6HG0503820 | Forward:TATGATATGGAGACCACCTACC | 1R_vs_1S_mock | 0.80 | 1.00 |
|  | Reverse:TTAGTAGTCATCTTCGTCGTCC | 2R_vs_2S_mock | 0.99 | 0.99 |
| HORVU.MOREX.r2.6HG0503840 | Forward:TGCACTGTTGTTTCGAACTGG | Fp_vs_mock_3R | 2.06 | 2.14 |
|  | Reverse:CCAAGGAGAACTCTTGTCATC |  |  |  |

Table S2. Number of differentially expressed genes (DEGs) identified from all pairwise comparisons.

| NIL pair | Comparison | Number of DEGs | |
| --- | --- | --- | --- |
|  |  | Up-regulated | Down-regulated |
| 6HL_NIL 1 | R^M^_vs_R^I^ | 692 | 160 |
|  | S^M^_vs_S^I^ | 746 | 67 |
| 6HL_NIL 2 | R^M^_vs_R^I^ | 296 | 129 |
|  | S^M^_vs_S^I^ | 420 | 117 |
| 6HL_NIL 3 | R^M^_vs_R^I^ | 640 | 359 |
|  | S^M^_vs_S^I^ | 280 | 44 |
| 6HL_NIL 1 | R^I^_vs_S^I^ | 400 | 305 |
|  | R^M^_vs_S^M^ | 582 | 254 |
| 6HL_NIL 2 | R^I^_vs_S^I^ | 418 | 353 |
|  | R^M^_vs_S^M^ | 633 | 279 |
| 6HL_NIL 3 | R^I^_vs_S^I^ | 308 | 170 |
|  | R^M^_vs_S^M^ | 258 | 118 |

Table S3. High- and low-confident genes in the genomic region harbouring *Qcrs.caf-6H.*

| Predicted gene ID^a^ | Physical location on  6H pseudomolecule (bp) | Confidence class^b^ | Annotation |
| --- | --- | --- | --- |
| HORVU.MOREX.r2.6HG0503660 | 472,315,109-472,316,773 | HC | Pimeloyl-[acyl-carrier protein] methyl ester esterase |
| HORVU.MOREX.r2.6HG0503680 | 472,581,748-472,584,057 | HC | Tubby-like F-box protein |
| HORVU.MOREX.r2.6HG0503690 | 472,585,926-472,589,193 | HC | Adenine nucleotide alpha hydrolases-like superfamily protein |
| HORVU.MOREX.r2.6HG0503700 | 472,589,578-472,591,434 | HC | Basic helix-loop-helix transcription factor |
| HORVU.MOREX.r2.6HG0503710 | 472,609,074-472,609,350 | LC | Retrovirus-related Pol polyprotein from transposon TNT 1-94 |
| HORVU.MOREX.r2.6HG0503720 | 472,863,603-472,864,529 | LC | WRKY DNA-binding protein 57 |
| HORVU.MOREX.r2.6HG0503730 | 472,864,835-472,867,986 | HC | U-box domain-containing family protein |
| HORVU.MOREX.r2.6HG0503740 | 472,868,519-472,868,908 | HC | Nodulin MtN21 /EamA-like transporter family protein |
| HORVU.MOREX.r2.6HG0503750 | 472,869,544-472,869,939 | HC | Receptor like protein 39 |
| HORVU.MOREX.r2.6HG0503760 | 472,960,476-472,960,781 | LC | DNA gyrase subunit B |
| HORVU.MOREX.r2.6HG0503770 | 472,962,662-472,966,016 | LC | Retrotransposon protein, putative, Ty3-gypsy subclass |
| HORVU.MOREX.r2.6HG0503780 | 472,969,893-472,970,702 | LC | Transcription regulator NOT2/NOT3/NOT5 family protein |
| HORVU.MOREX.r2.6HG0503790 | 472,973,258-472,974,388 | LC | LINE-1 retrotransposable element ORF2 protein |
| HORVU.MOREX.r2.6HG0503800 | 472,974,445-472,975,671 | LC | RNA-directed DNA polymerase (reverse transcriptase)-related family protein |
| HORVU.MOREX.r2.6HG0503810 | 472,977,409-472,979,100 | HC | Transcription factor RADIALIS |
| HORVU.MOREX.r2.6HG0503820 | 473,074,179-473,077,165 | HC | Chromatin modification-related protein MEAF6 |
| HORVU.MOREX.r2.6HG0503830 | 473,084,019-473,086,919 | LC | UvrABC system protein B |
| HORVU.MOREX.r2.6HG0503840 | 473,127,996-473,132,106 | HC | ATPase family AAA domain-containing protein 3 |

^a^ The predicted genes and their annotations were retrieved from IPK-Gatersleben blast-website (https://webblast.ipk-gatersleben.de/barley_ibsc/downloads/). Genes in red contain SNP variations between resistant and susceptible alleles.

^b^ The confidence classes include *HC,* high-confidence gene and *LC*, low-confidence gene.

Table S4. SNPs detected between R and S isolines for the genes located in the interval harbouring the *Qcrs.caf-6H* locus and their expression levels.

| Gene ID | Annotation | SNP Position | R allele | S allele | Substitution type | Amino acids variations | Differential expression (Log2FC) |
| --- | --- | --- | --- | --- | --- | --- | --- |
| HORVU.MOREX.r2.6HG0503660 | Pimeloyl-[acyl-carrier protein] methyl ester esterase | 472585943 | A | G | synonymous_variant | Glu6Glu | 2R_vs_2S_mock (-1.97) |
|  |  | 472586470 | GTAATAATA | GTAATA | downstream_gene_variant |  |  |
|  |  | 472586566 | C | G | downstream_gene_variant |  |  |
|  |  | 472586629 | C | G | downstream_gene_variant |  |  |
|  |  | 472586660 | A | G | downstream_gene_variant |  |  |
|  |  | 472586925 | A | G | downstream_gene_variant |  |  |
|  |  | 472587113 | T | A | downstream_gene_variant |  |  |
|  |  | 472587226 | T | C | downstream_gene_variant |  |  |
| HORVU.MOREX.r2.6HG0503700 | Basic helix-loop-helix transcription factor | 472590558 | A | C | downstream_gene_variant |  | 1R_vs_1S_mock (-2.51) |
|  |  | 472590570 | A | G | downstream_gene_variant |  | 3R_vs_3S_Fp (-2.17) |
|  |  | 472590614 | T | C | downstream_gene_variant |  |  |
|  |  | 472590618 | G | A | downstream_gene_variant |  |  |
|  |  | 472591038 | C | T | synonymous_variant | Pro105Pro |  |
| HORVU.MOREX.r2.6HG0503730 | U-box domain-containing family protein | 472865385 | G | A | missense_variant | Ser587Leu | Fp_vs_mock_1S (-2.10) |
|  |  | 472866181 | C | A | missense_variant | Ala376Ser | Fp_vs_mock_2S (-1.90) |
|  |  | 472866592 | T | A | missense_variant | Ser239Cys |  |
| HORVU.MOREX.r2.6HG0503820 | Chromatin modification-related protein MEAF6 | 473074729 | T | C | intron_variant |  |  |
|  |  | 473076868 | A | G | intron_variant |  |  |
| HORVU.MOREX.r2.6HG0503840 | ATPase family AAA domain-containing protein 3 | 473130339 | T | A | synonymous_variant | Gly322Gly | Fp_vs_mock_3R (2.06) |


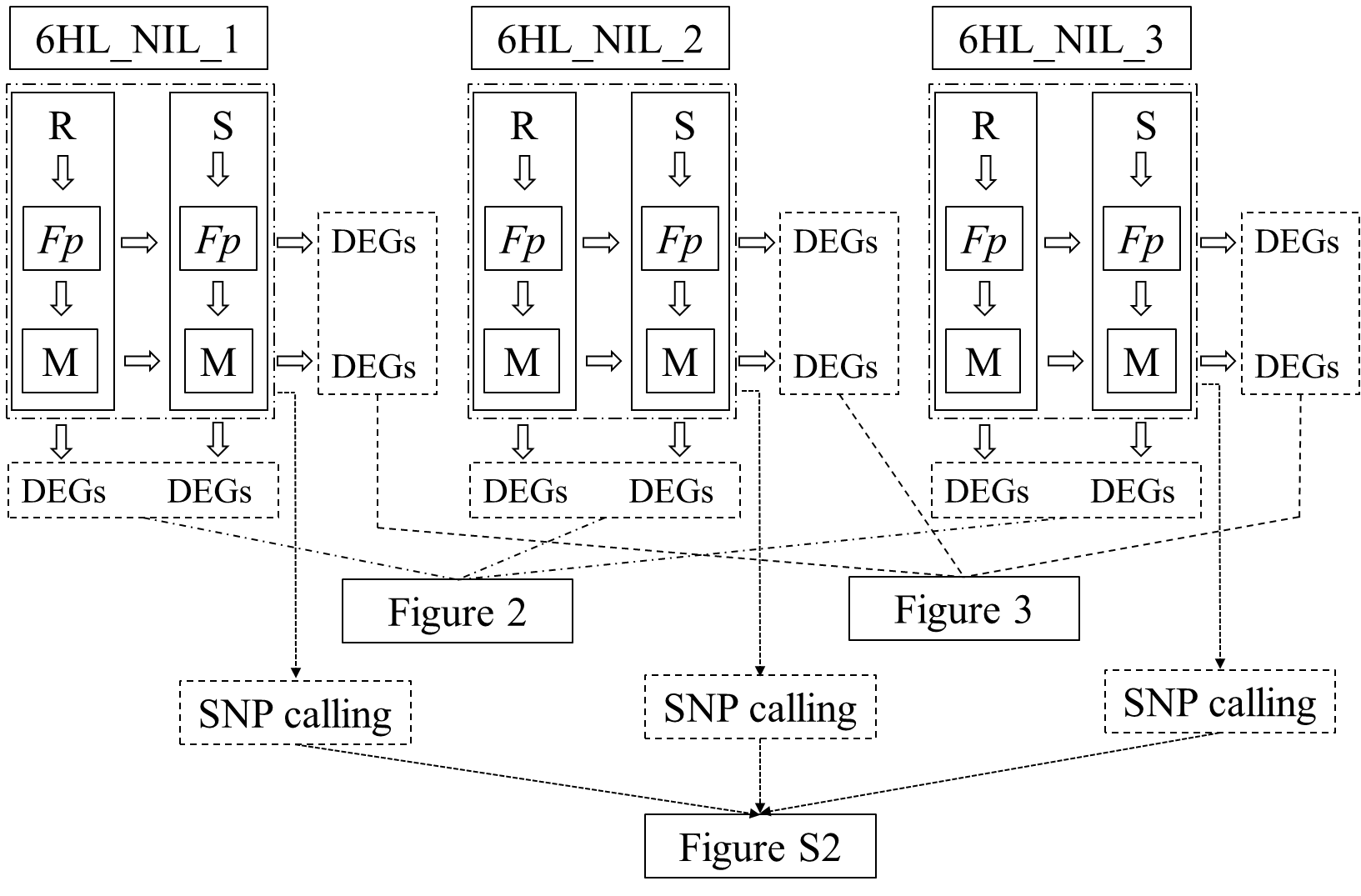


**Figure S1.** Schematic showing the experimental design for differential gene expression analysis and SNP calling.


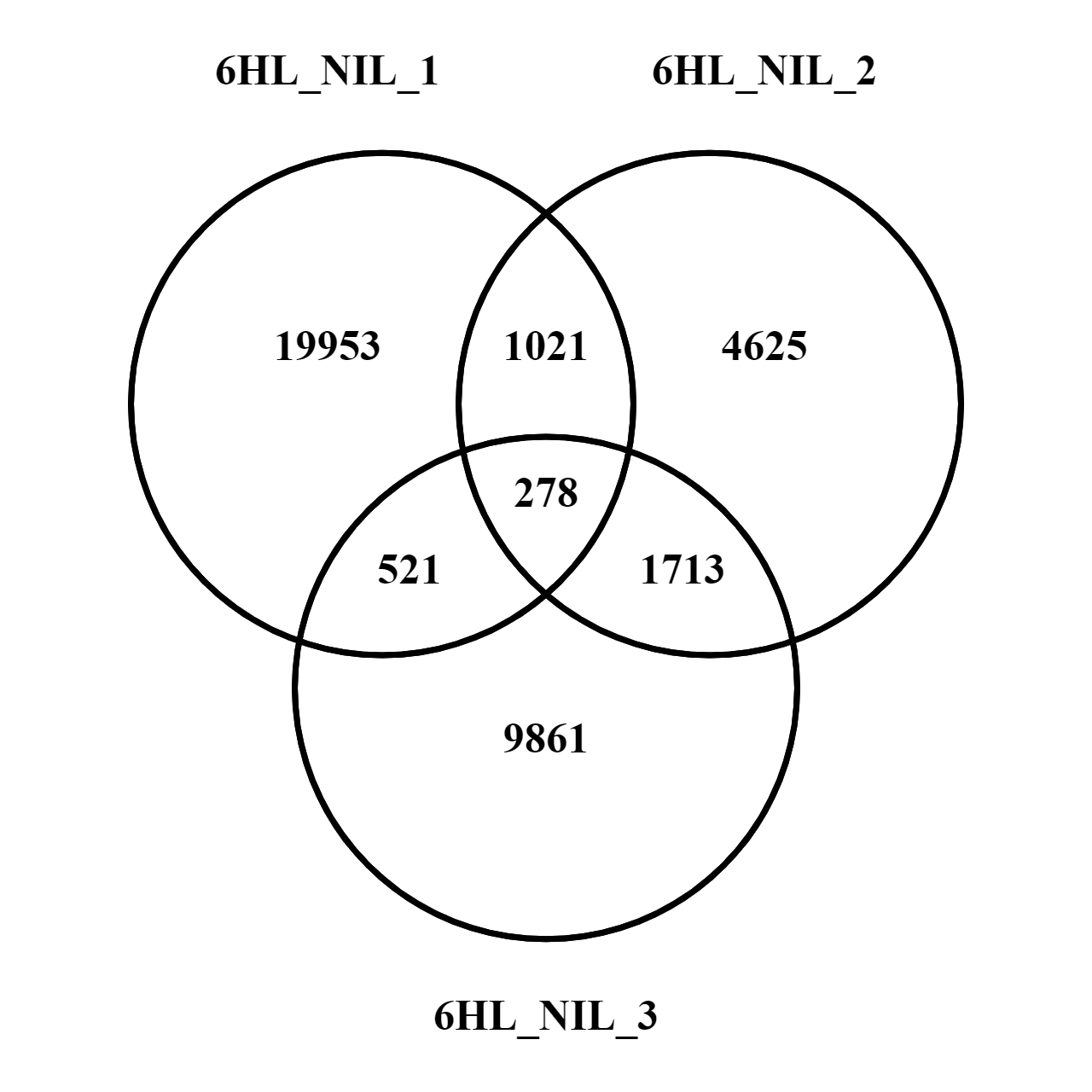


**Figure S2.** Numbers of SNPs detected between R and S isolines. A Venn diagram showing the numbers of unique and common SNPs between resistant and susceptible isolines among three NIL pairs.


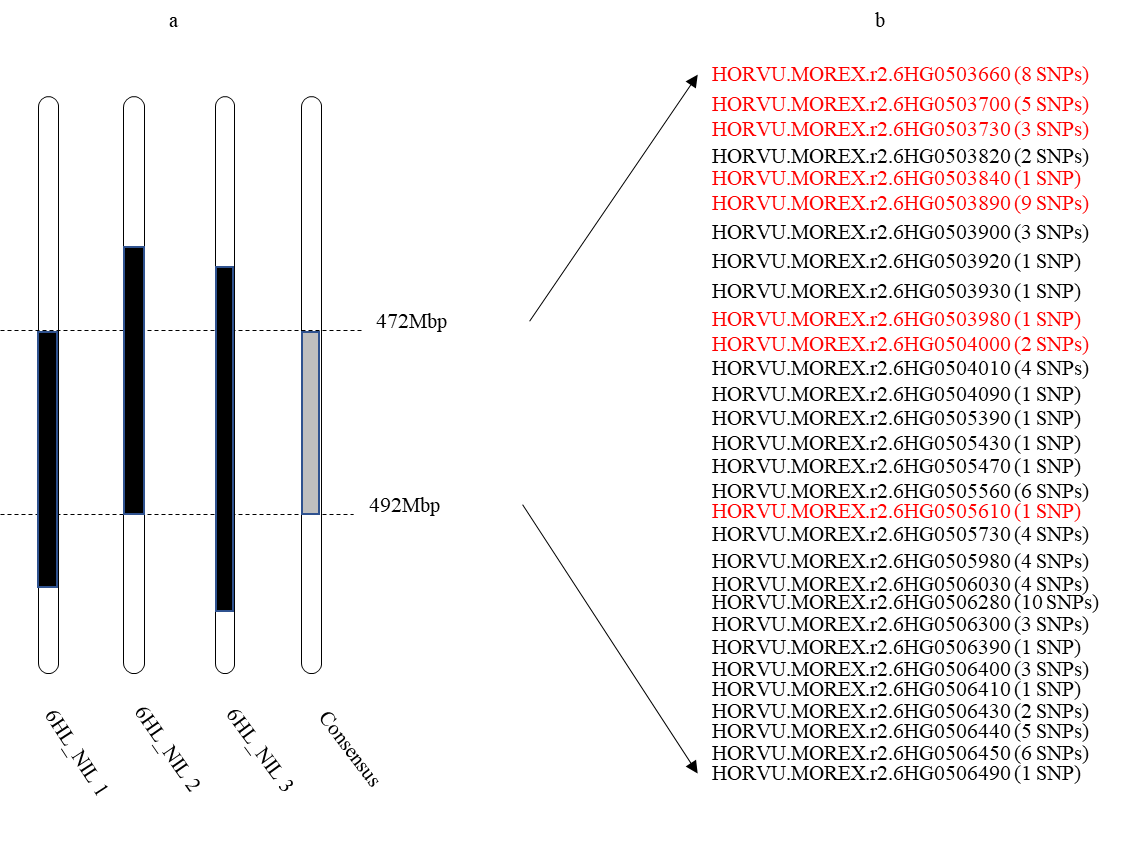


**Figure S3.** High confident genes identified within the consensus SNP-enriched region. **a** The physical range of SNP-enriched region. Black boxes indicate the regions defined by SNPs within each 6HL-NIL pair; the grey box represents the consensus region. **b** High confident genes containing the SNPs among the three comparisons within the consensus region. The numbers of SNPs identified within genes were in brackets and DEGs detected between isolines for at least one of the NIL pairs in the region were marked in red.


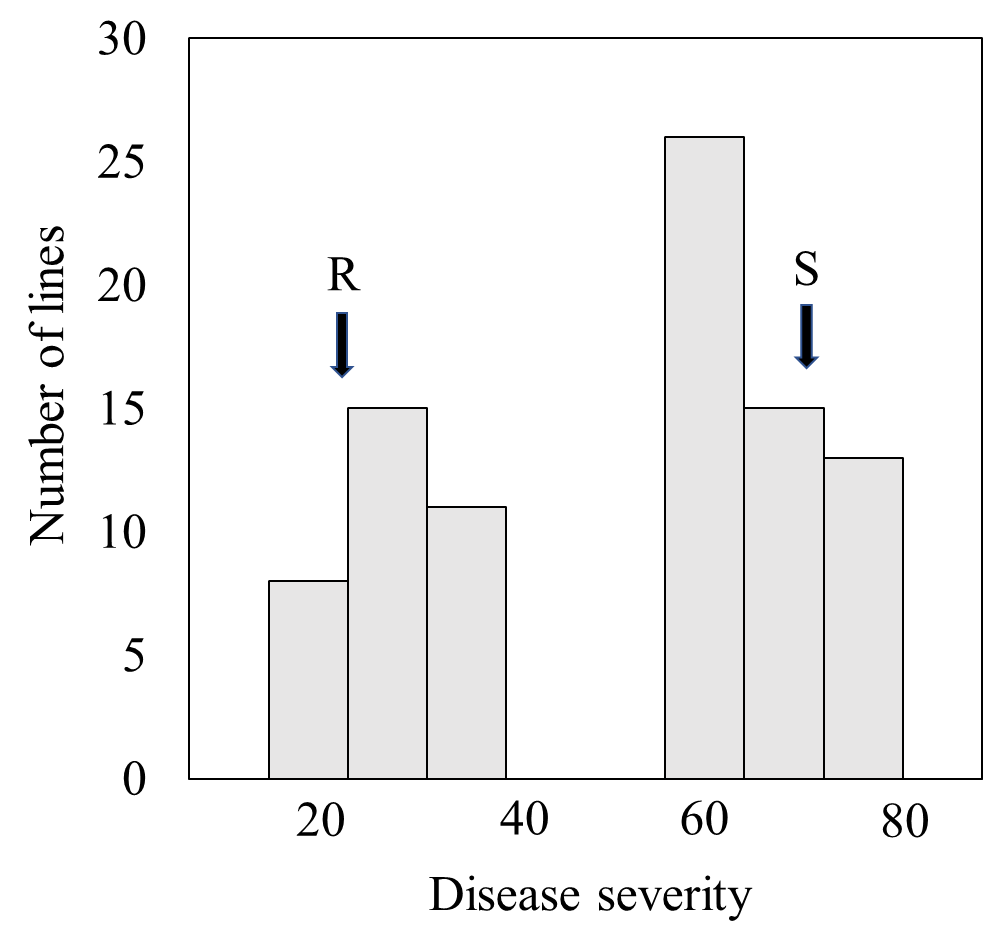


**Figure S4.** Distribution of FCR severity in the subpopulation of 88 lines. Disease severities of the two controls (NIL_CR6HL_1R and NIL_CR6HL_1S) were indicated with black arrows.
